# Genome-wide identification and abiotic stress response analysis of the oat *bHLH* gene family

**DOI:** 10.3389/fpls.2026.1832370

**Published:** 2026-04-20

**Authors:** Huajuan Leng, Xiaopeng Zhang, Tian Zhang, Jing Cui, Yangyang Zhang, Hongbo Dai, Lili Zhang, Huancheng Liu, Xue Wang, Junmei Kang, Minna Zheng, Yue Li, Tiejun Zhang

**Affiliations:** 1 Institute of Animal Science, Chinese Academy of Agricultural Sciences, Beijing, China; 2 School of Grassland Science, Beijing Forestry University, Beijing, China; 3 High Latitude Crops Institute, Shanxi Agricultural University, Datong, China; 4 College of Grassland Science and Technology, China Agricultural University, Beijing, China

**Keywords:** abiotic stress, *Avena sativa L*., bHLH gene family, transcription factor, whole genome analysis

## Abstract

**Introduction:**

Basic helix-loop-helix (bHLH) transcription factors constitute a major gene family that governs plant development and abiotic stress responses. However, no comprehensive study of this family has been reported in hexaploid oat (*Avena sativa* L.).

**Methods:**

We performed genome-wide identification and characterization of the *bHLH* family in oat, and delineated 271 *AsbHLH* genes unevenly distributed across the genome. Phylogenetic and evolutionary analyses were conducted, followed by expression profiling under salt, drought, gibberellin, methyl jasmonate, and salicylic acid treatments. Functional validation was carried out using an *Arabidopsis bhlh257* mutant, an ortholog of a differentially expressed oat gene.

**Results:**

Phylogenetic and evolutionary analyses revealed a conserved subfamily structure alongside lineage-specific features, with segmental duplication identified as the primary driver of family expansion. Expression profiling identified a subset of core candidates, including *AsbHLH110*, *AsbHLH121*, *AsbHLH204*, and *AsbHLH257*, that were co-responsive to all tested stresses and hormones. Notably, *AsbHLH257* demonstrated a 12.9-fold induction under drought stress (p< 0.0001). Functional validation using the *Arabidopsis bhlh257* mutant confirmed the conserved role of *bHLH* transcription factors in drought tolerance and linked them to the regulation of oxidative stress homeostasis.

**Discussion:**

The co-responsive pattern supports the potential role of these candidates as integrative nodes in stress-hormone signaling networks. Collectively, this work establishes a prioritized candidate gene resource with crucial functional insights, providing a foundation for targeted genetic improvement of stress resilience in oat.

## 1 Introduction

The basic helix-loop-helix (bHLH) proteins constitute one of the largest superfamilies of transcription factors in eukaryotes, playing pivotal roles in regulating diverse plant processes, including growth, development, and environmental responses (Heim et al., 2003; Carretero-Paulet et al., 2010). The characteristic bHLH domain, approximately 60 amino acids in length, comprises two functionally distinct regions: an N-terminal basic region responsible for DNA binding to the E-box motif (CANNTG) and a C-terminal helix-loop-helix (HLH) region that mediates protein dimerization (Toledo-Ortiz et al., 2003; Ellenberger et al., 1994). This conserved architecture facilitates the formation of homodimeric or heterodimeric complexes, serving as a central mechanism for transcriptional regulation. In addition to the core domain, sequence variation and additional conserved motifs contribute to the functional diversification observed among bHLH subfamilies (Pires and Dolan, 2010).

The *bHLH* family underwent significant expansion early in land plant evolution, a process believed to have contributed to enhanced environmental adaptability (Pires and Dolan, 2010). This evolutionary expansion is tightly linked to the well-documented role of the family in mediating abiotic stress tolerance. For instance, the *Arabidopsis* transcription factor phytochrome-interacting factor 3 (PIF3) integrates light and temperature signals to regulate thermomorphogenesis (Das et al., 2025), highlighting the role of *bHLH*s in signal integration. Numerous other members are key mediators of specific stress responses: *AtbHLH92* and *AtbHLH112* confer tolerance to salt and drought stress, respectively (Jiang et al., 2009; C. Li et al., 2021), while in crops, *CsbHLH041* (cucumber) and *MdbHLH130* (apple) modulate salt/ABA responses and drought tolerance (J. Li et al., 2020; Zhao et al., 2020). Notably, several of these adaptive responses involve crosstalk between bHLH factors and phytohormone signaling pathways such as abscisic acid (ABA), jasmonic acid, and salicylic acid (Feller et al., 2011; Qiu et al., 2020). This positions *bHLH* transcription factors as crucial nodes in the network that integrates environmental cues with hormonal regulation to coordinate plant adaptation.

Systematic analyses of the *bHLH* gene family in model plants such as *Arabidopsis* and rice have established a framework for understanding its roles in growth and environmental adaptation (Li et al., 2006; Toledo-Ortiz et al., 2003). Cross-species comparisons have further revealed patterns of gene family evolution and functional diversification, highlighting both conserved and lineage-specific traits (Carretero-Paulet et al., 2010; Pires and Dolan, 2010). Although the *bHLH* gene family has been studied in several plant species, including *Arabidopsis*, rice, maize, and wheat, limited data are currently available for oats, an important and unique cereal crop. The oat genome was recently sequenced and published; however, no comprehensive study of the *bHLH* gene family in oats has yet been reported. Therefore, in the present study, we aimed to systematically identify and characterize the *bHLH* gene family in oats and compare its members with those of rice and *Arabidopsis*. We also aimed to analyze the *AsbHLH* gene expression across different tissues under abiotic stress conditions. Therefore, we collectively aimed to perform a genome-wide identification and characterization of the *bHLH* gene family in oat. To provide an initial functional context, we conducted a case study examining the expression of three selected *AsbHLH* genes (*AsbHLH110*, *AsbHLH121*, and *AsbHLH204*) under a range of abiotic stresses and hormone treatments. The results showed that all three genes responded to varying degrees under both salt and drought stress, and were modulated by multiple hormones, providing a proof of concept and identifying specific, promising candidates for follow-up investigations.

## 2 Materials and methods

### 2.1 Gene identification

The protein sequences of the oat genome were obtained from the GrainGenes database | A Database for *Triticeae* and *Avena* (usda.gov). To achieve a comprehensive and unbiased identification of *bHLH* transcription factors, a conservative two-step strategy was implemented. First, all possible bHLH proteins were identified from the oat sequences (*Avena sativa* L.) using the Basic Local Alignment Search Tool for proteins (BLASTp). A permissive E-value cutoff of 1e-10 was applied in this initial phase to maximize sensitivity and compile a broad candidate pool. Subsequently, stringent domain validation was performed to confirm family membership. All candidate sequences were analyzed for the presence of the definitive bHLH domain (PF00010) using Hidden Markov Models for Efficient Requirement (HMMER) 3.0 (http://plants.ensembl.org/hmmer/index.html) with a significant E-value threshold of ≤ 1e-5. The domain architecture was further verified through manual inspection using the Simple Modular Architecture Research (SMART) database (http://smart.emblheidelberg.de/). Only sequences that passed both the HMMER and SMART validations were retained as confident members of the oat bHLH (*AsbHLH*) family.

### 2.2 Gene structure of *bHLHs*

ClustalW, with default parameters (K-tuple size: 1, Window size: 5, Gap Penalty: 3, Number of Top Diagonals: 5, Scoring Method: PERCENT), was used to perform multiple protein sequence alignments between the 271 *AsbHLH* genes and *Arabidopsis* bHLH proteins. GeneDoc 2.7 software was used to manually adjust the deduced amino acid sequences of the bHLH domain. Using the genome annotation GFF file, the Gene Structure Display Server (http://GSDS.cbi.pku.edu.cn) was used to visualize the exon/intron structure of each *AsbHLH* gene. Next, the bHLH protein motifs were identified using the MEME server (http://meme-suite.org/tools/meme) with the following settings: 10 motifs and 6–200 residues, and the commonly adopted site distribution model “Zero or One Occurrence Per Sequence (zoops)”.

### 2.3 Chromosome distribution and gene duplication

Using Circos and the oat genome database (https://plants.ensembl.org/index.html), all *AsbHLH* genes were mapped to 22 chromosomes and Chr00 of *Avena sativa*. Gene duplication events were analyzed using MCScanX software with default parameters (CPU for BLASTP: 2; E-value: 1e-10; Num of BLASTHits: 5). We used the duel synteny plot of TBtools (https://github.com/CJ-Chen/TBtools) to analyze homology within the two species (*A. thaliana* and *O. sativa*). Non-synonymous (Ka) and synonymous (Ks) substitution rates for each duplicated *bHLH* gene were calculated using Ka/Ks Calculator 2.0.

### 2.4 Phylogenetic analysis and classification of the *AsbHLH* gene family

All identified *AsbHLH* genes were classified based on the known bHLH transcription factor groups in *A. thaliana*. A neighbor-joining (NJ) phylogenetic tree was constructed using the Jukes–Cantor model in MEGA 7.0 with 1000 bootstrap replicates. The BLOSUM62 cost matrix was used to determine genetic relationships. Protein sequences of *A. thaliana* (dicot) and *O. sativa* (monocot) were obtained from the UniProt database (https://www.uniprot.org/) and relevant publications (Wei and Chen, 2018), and were combined with the 271 AsbHLH sequences for phylogenetic analysis. The subgroup assignment for each AsbHLH protein was determined primarily by its placement within the phylogenetic tree. Specifically, an AsbHLH protein was assigned to a specific *Arabidopsis* subgroup if it clustered within a well-supported monophyletic clade (bootstrap value ≥ 70%) that was predominantly composed of members from that *Arabidopsis* subgroup. This classification followed the established framework for *Arabidopsis* bHLH proteins. For genes that fell into poorly supported branches (bootstrap < 70%), their classification was further verified by examining the composition and order of conserved protein motifs (as identified by MEME analysis) against the characteristic motif patterns of the *Arabidopsis* subgroups.

### 2.5 Plant materials, growth conditions, and abiotic stresses

Common oat (*A. sativa* L., 2n = 6x = 42; AACCDD genome) is one of the most recently sequenced species. Oat plants (Zhongyan No.4; The seeds were provided by Institute of Crop Science, Chinese Academy of Agricultural Sciences.) were cultivated at the Institute of Animal Science, Chinese Academy of Agricultural Sciences (CAAS), Beijing, China, in a growth room maintained at 75% relative humidity under a 16-h light (25 °C)/8-h dark (20 °C) photoperiod. When the plants reached the seedling stage (day 35), five uniformly grown samples under similar conditions were selected. Roots, stems, and leaves were collected and immediately frozen in liquid nitrogen at –80 °C for preservation.

Five abiotic stress treatments were applied: salicylic acid (15% SA), methyl jasmonate (15% MeJA), gibberellic acid (200 mmol/L GA), drought stress (15% PEG solution), and salt stress (200 mmol/L NaCl). Leaf samples were collected at 0, 3, 6, 12, and 24 h after treatment and immediately frozen in liquid nitrogen at –80 °C for storage.

### 2.6 Total RNA extraction, cDNA synthesis, and qRT-PCR analysis

Total RNA was extracted using a plant RNA extraction kit (TaKaRa Bio) and treated with DNase I (without RNase) to eliminate residual genomic DNA. qRT-PCR primers were designed using Primer Premier 5.0, and the primer sequences are listed in **Supplementary Table 1** using Primer Premier 5.0. *GAPDH* was used as an internal control. qRT-PCR was performed in triplicate using SYBR Premix Ex Taq II (TaKaRa Bio) with a sample size of n = 3. We used 2^-ΔΔCt^ to calculate relative gene expression. Statistical analyses were performed using GraphPad Prism 10.4.1 software, and differences were determined by one-way analysis of variance (one-way ANOVA) followed by Dunnett’s multiple comparison test.

### 2.7 Drought stress treatment and physiological assays

Seeds of *A. thaliana* WT and the *bhlh257* mutant (At5g48560) were surface-sterilized, stratified at 4 °C for 3 days, and germinated on half-strength Murashige and Skoog (1/2 MS) medium. Seedlings at 12 days of age were transplanted into vermiculite soil, and grown under well-watered conditions for 21 days. At this point, on stabilization of soil moisture content at approximately 70% (v/v), watering was completely ceased for 14 days in order to induce drought stress. Histochemical analysis was performed by incubating detached leaves in nitro blue tetrazolium (NBT) solution for superoxide anion (O_2_^-^) detection, and in 3,3’-diaminobenzidine (DAB) solution for hydrogen peroxide (H_2_O_2_) visualization, followed by decolorization in ethanol. The hydrogen peroxide (H_2_O_2_) and malondialdehyde (MDA) levels were quantified using commercial assay kits (Hydrogen Peroxide assay kit, Nanjing Jiancheng Bioengineering Institute, A064-1-1; Malondialdehyde assay kit, Nanjing Jiancheng Bioengineering Institute, A003-1-2). The activities of superoxide dismutase (SOD) and catalase (CAT) were determined using commercial assay kits (Total Superoxide Dismutase assay kit, Nanjing Jiancheng Bioengineering Institute, A001-1-2; Catalase assay kit, Nanjing Jiancheng Bioengineering Institute, A007-1-1) as per the manufacturer’s protocols. Data are presented as mean ± standard deviation (SD) of three independent biological replicates. Statistical analyses were conducted using GraphPad Prism 10.4.1 software. Differences between WT and the *bhlh257* mutant under the same treatment condition (i.e., within the control group or within the drought-treated group) were determined using the one-way analysis of variance (ANOVA), followed by Dunnett’s multiple comparison test.

## 3 Results

### 3.1 Identification of *bHLH* genes in oats (*A. sativa* L.)

All putative bHLH family members in the oat genome were identified using two structural domain recognition methods. To distinguish these genes, we designated them *AsbHLH001*–*AsbHLH271* based on their chromosomal locations (**Supplementary Table S1**), along with associated data on amino acid number, Mw, pI, CDS, domain structure, and predicted subcellular localization. Among the 271 proteins, AsbHLH232 was the smallest, comprising 131 amino acids, whereas AsbHLH191 was the largest, containing 1168 amino acids. The average amino acid length was 383. The molecular weight of the 271 AsbHLHs ranged from 14.43 kDa (AsbHLH232) to 132.08 kDa (AsbHLH191), with an average of 41.27 kDa. The pI values ranged 4.5 (AsbHLH079) to 10.54 (AsbHLH168), with a mean of 6.74. Based on predicted subcellular localization, 236 AsbHLH proteins were located in the nucleus, 29 in chloroplasts, one in the cytoplasm (AsbHLH221), one in the Golgi (AsbHLH150), two in mitochondria (AsbHLH018 and AsbHLH148), and two in peroxisomes (AsbHLH085 and AsbHLH108).

**Figure 1:**
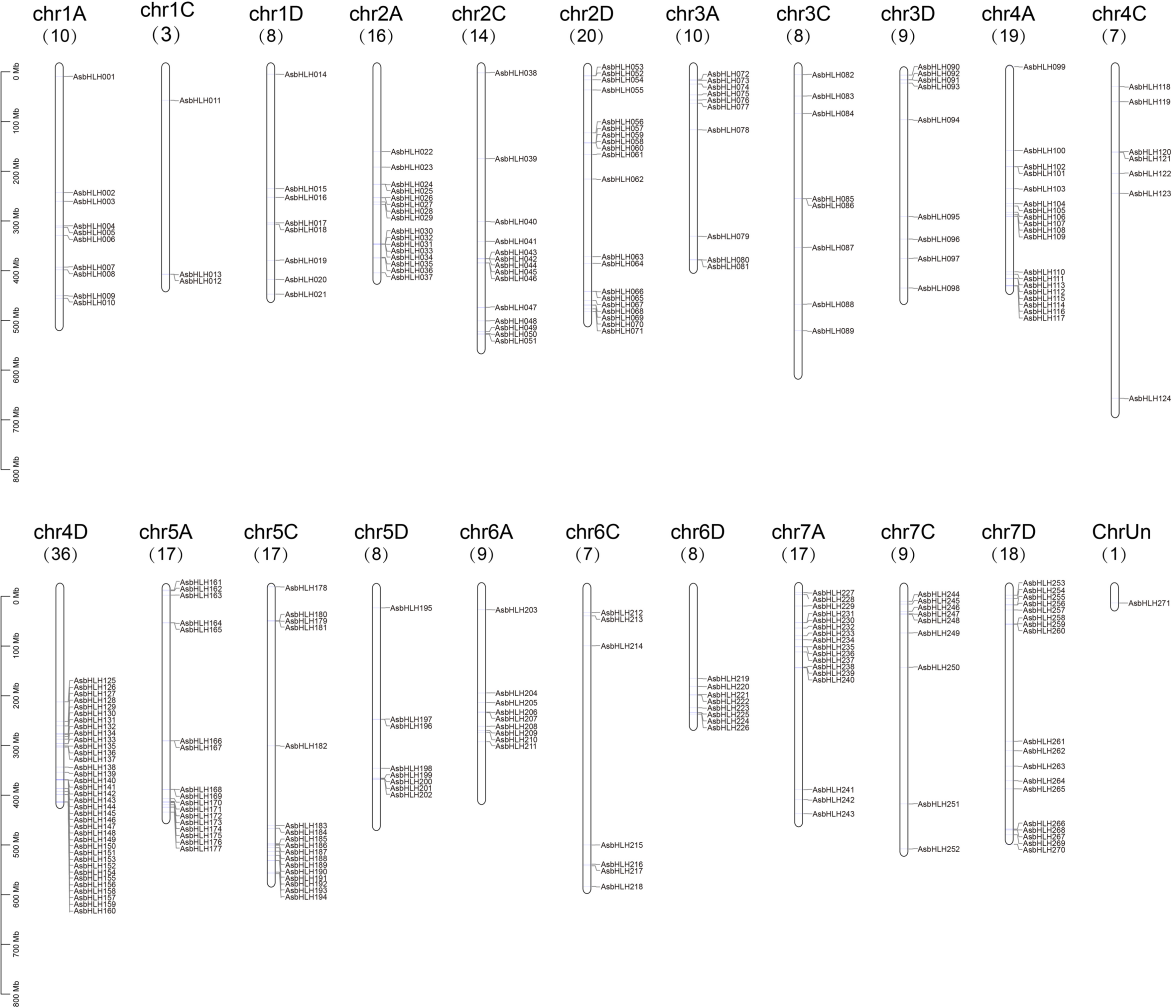
The distribution of *bHLH* genes on oat chromosomes. *AsbHLHs* are located on 21 chromosomes and named according to its physical location. *AsbHLH271* on ChrUn is a gene not located on Chr1-Chr7.

A chromosomal distribution map of the *AsbHLH* genes was constructed (**Figure 1**). Among the 270 identified genes, the majority were unevenly dispersed across all 21 chromosomes, whereas one remained unassigned to any specific chromosome. The genes were systematically designated based on their sequential physical positions from the top to the bottom of each chromosome (Chr1A to Chr7C). Chromosomal distribution analysis showed that Chr4D harbored the highest number of genes (36, 13.28% of the family), followed by Chr2D (20, 7.38%), whereas Chr1C contained the fewest genes (3, 1.10%).

### 3.2 Multiple sequence alignment, phylogenetic analysis, and classification of AsbHLH proteins

**Figure 2:**
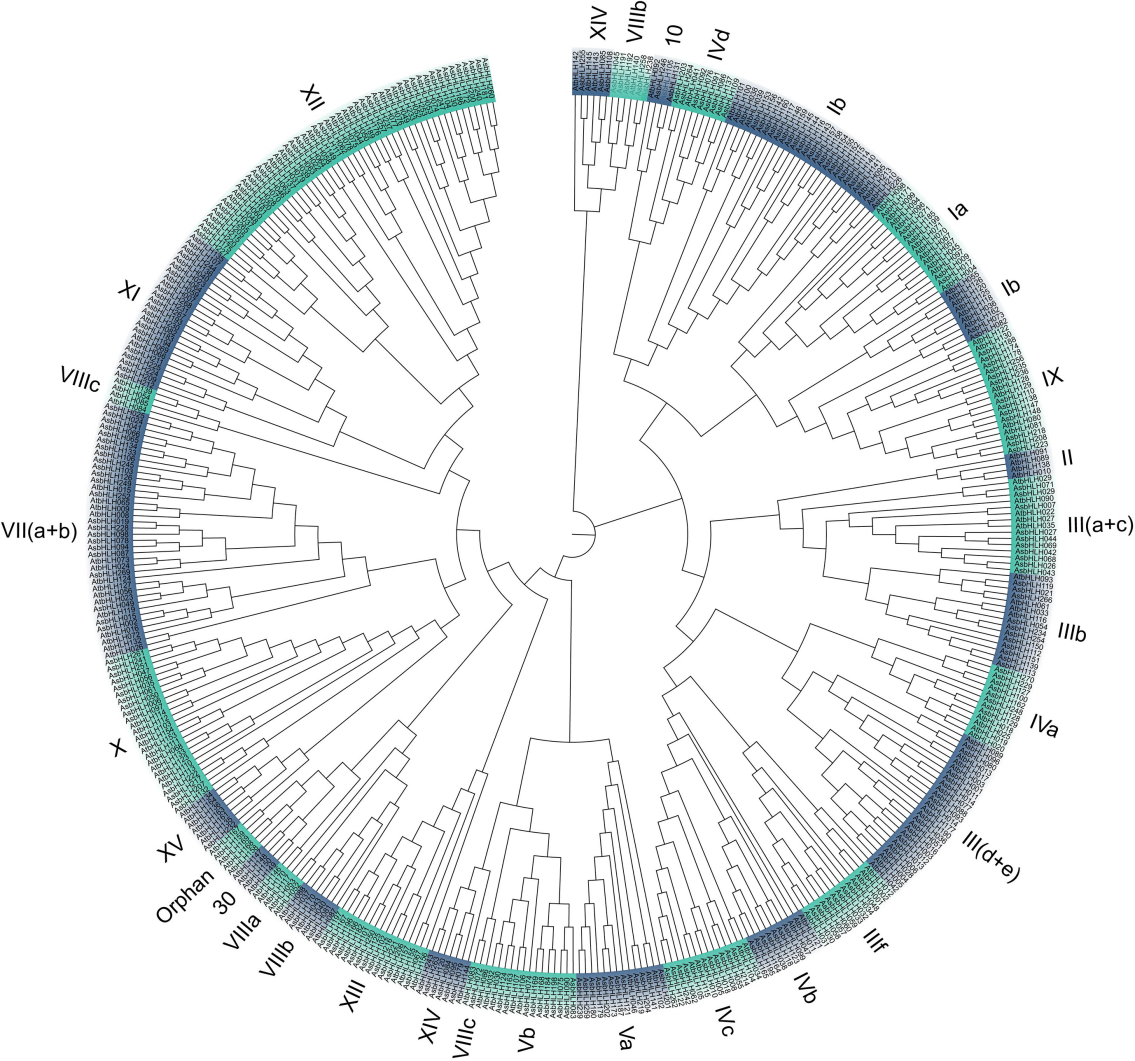
Phylogenetic tree constructed of bHLH proteins from oat and *Arabidopsis*. The peripheral Roman numerals represent the subgroup grouping, and the adjacent subgroups are marked with blue and green intervals. Two colors are used alternately to distinguish different groups.

Using the 271 identified AsbHLH and 161 *Arabidopsis* bHLH proteins, we constructed a phylogenetic tree using the neighbor-joining tree method with 1000 bootstrap replicates (**Figure 2**). Phylogenetic analysis of the 271 AsbHLH proteins, based on tree topology and the Pires and Toledo Ortiz classification system, resolved them into 26 distinct subgroups. This organization mirrors the established subgrouping of *Arabidopsis* bHLH proteins, thus supporting their evolutionary conservation. Notably, subgroup 20 was notably absent in the oat *bHLH* family, suggesting either evolutionary loss or lack of differentiation during oat speciation. Among the 26 subgroups, subgroup XII was the largest (34 AsbHLH members), whereas subgroups VIIIc and XV contained only one member each. Phylogenetic analysis revealed that several AsbHLH proteins clustered closely with AtbHLH proteins (bootstrap support ≥ 80). Covariance analysis between oat and *Arabidopsis* indicated that AsbHLH proteins share homology with AtbHLH proteins, indicating potential functional similarities.

### 3.3 Analysis of gene structure and conserved domains of AsbHLHs

**Figure 3:**
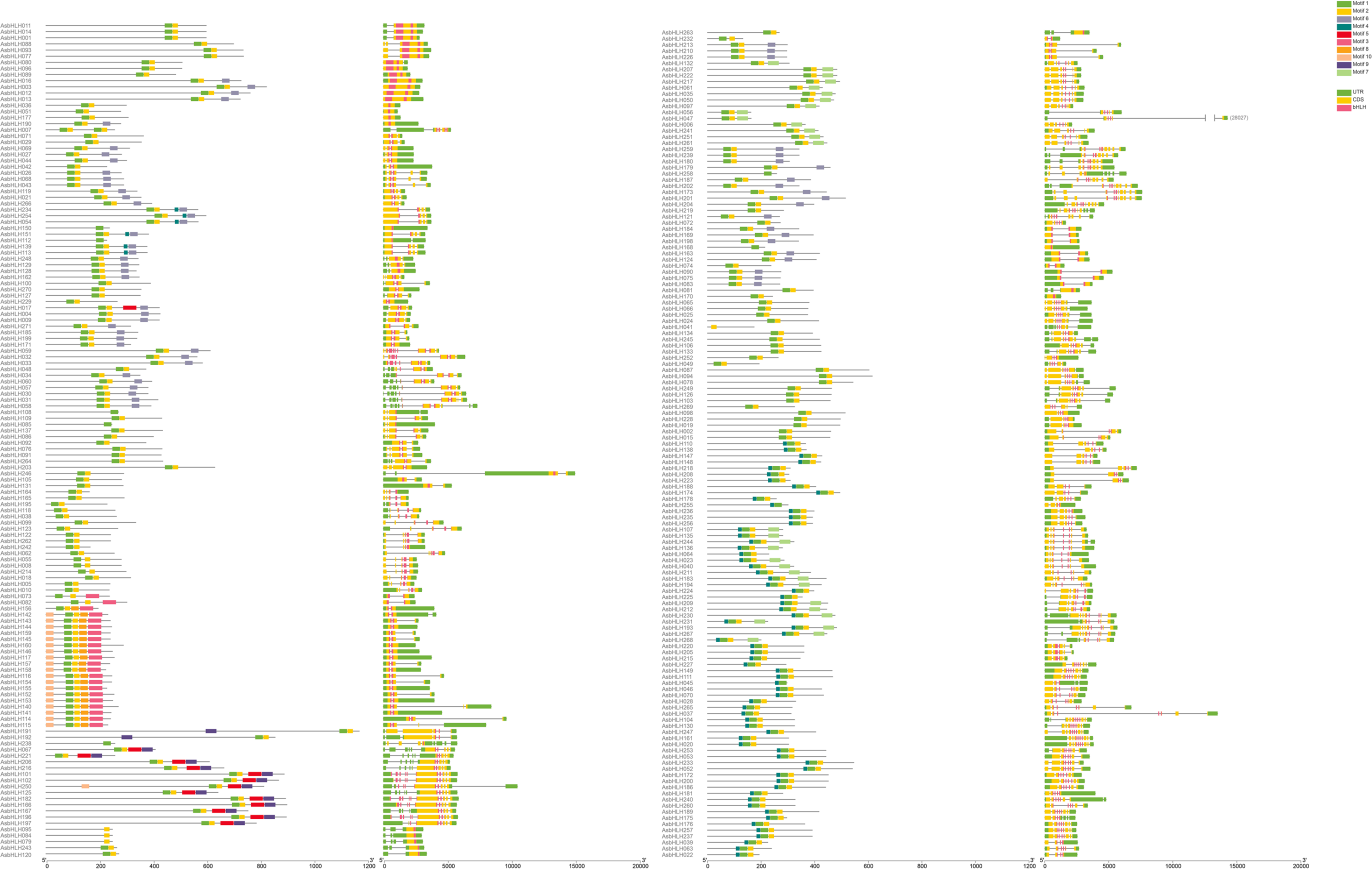
The gene structure and motif of AsbHLH*s*. Column 1, 3 and column 2, 4 diagrams are protein motif and gene structure, respectively. The arrangement follows the order of the Neighbor-joining phylogenetic tree of AsbHLH genes. Ten distinct colors represent different motifs. Green, yellow, and pink boxes denote exons, untranslated regions (UTRs), and the basic structure of AsbHLH genes, respectively, while black lines indicate introns.

All 271 identified *AsbHLH* genes in oat were observed to possess at least one intron (**Figure 3**). Notably, *AsbHLH201* (subgroup Va) exhibited the most complex gene structure with 11 introns, the highest number observed among all family members (**Supplementary Figure 1**; the individual phylogenetic tree of the AsbHLH family). These intronic sequences showed significant length variation, indicating potential functional diversity in their regulatory roles for gene splicing and expression.

**Figure 4:**
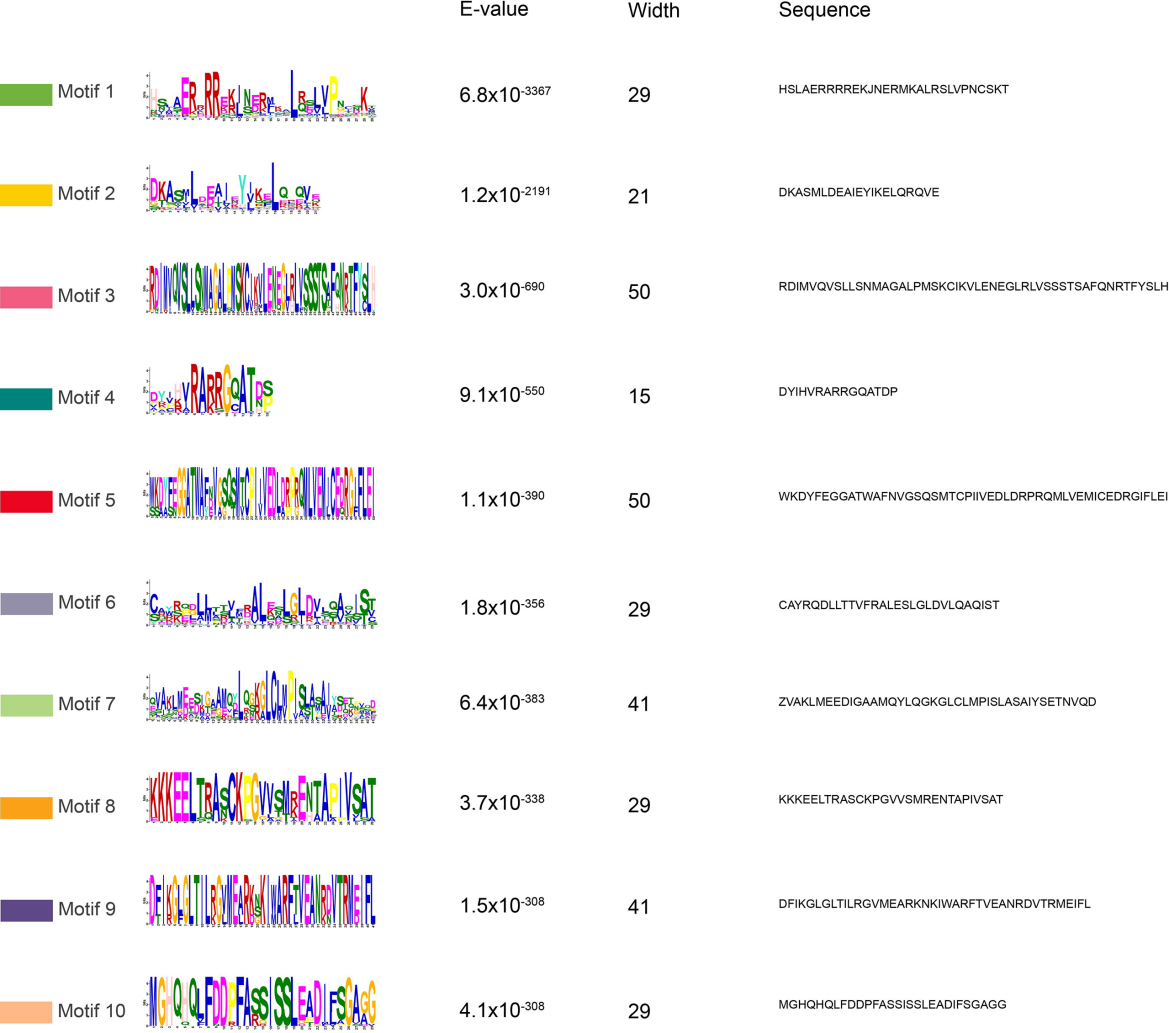
Conserved motif analysis of AsbHLHs. Prediction information of 10 conserved motifs of AsbHLHs. Visualization using MEME server.

For conserved domain analysis, MEME-based motif identification revealed ten characteristic motifs (designated Motif 1-Motif 10) distributed across the 271 *AsbHLH*s. This motif conservation pattern further supports the functional classification of these transcription factors (**Figures 3** and **4**). Most *AsbHLH* members contained Motifs 1 and 2. Among them, most of the seven *AsbHLH* members classified into subgroup Ia contained Motif 6, whereas the 25 members of subgroup Ib possessed Motifs 3, 8, and 10. Among the members classified in subgroups III(a+c), III(d+e), and IIIf, most of the 37 *AsbHLH* members contained Motif 6 in addition to Motifs 1 and 2. In contrast, the 11 *AsbHLH* members classified as IIIb contained Motifs 4 and 6. Among the eight members classified into subgroup IVa, some had Motif 6. Eleven *AsbHLH* members belonged to subgroup Va, and most contained Motifs 6 and 7. Ten *AsbHLH* members were classified into subgroup Vb, with most of them possessing Motif 6. Among the 19 *AsbHLH* members in subgroup XI, most had Motifs 4 and 7. A total of 34 *AsbHLH* members in subgroup XII predominantly contained Motif 4. A total of 13 *AsbHLH* members in subgroup XIII generally harbored Motifs 5 and 9. Eight *AsbHLH* members were classified into subgroup XIV, and some of them had only Motif 2.

These results indicate that Motifs 1 and 2 may comprise the conserved DNA-binding domain (DBD) and could serve as characteristic markers to determine whether a gene belongs to the *bHLH* gene family. In addition, some motifs were specific to particular subgroups or individual *AsbHLH* members. For instance, Motif 3 was exclusively observed in subgroup Ib, whereas Motifs 5 and 9 were restricted to subgroup XIII. These findings suggest that *AsbHLH* genes within the same subgroup possess similar structural and motif compositions, and that motif diversity may contribute to the functional diversity observed among *AsbHLH* members.

### 3.4 Gene duplication events and synteny analysis among *AsbHLH* genes

**Figure 5:**
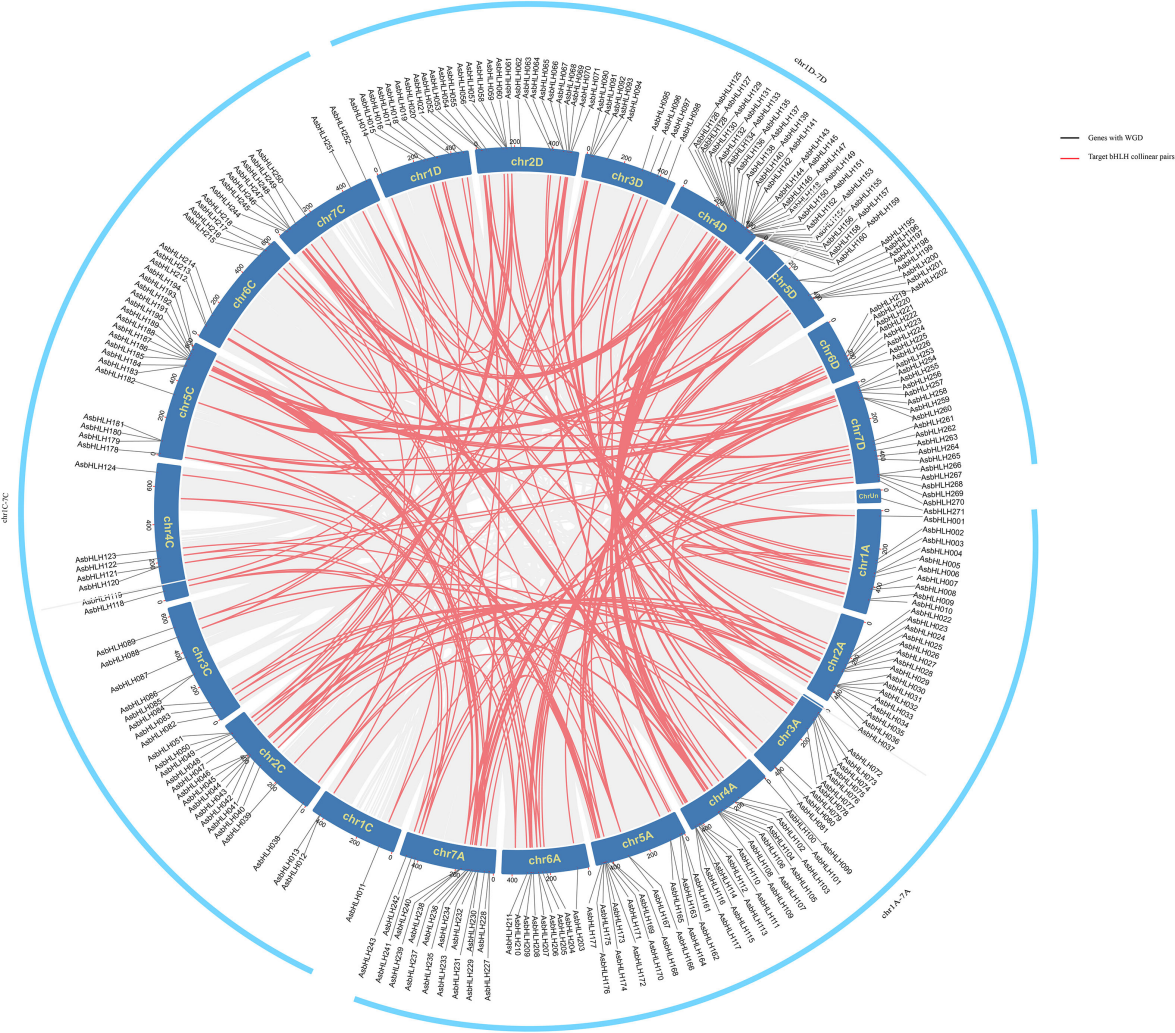
A schematic diagram of the *AsbHLH* gene in oat. Blue represents the chromosomes, gray represents the homologous region in the oat genome, and red represents the duplication event of the fragment. Grey lines signify genes that have undergone WGD, while red lines highlight collinear pairs of the target bHLH gene family.

To elucidate the evolutionary expansion of the *AsbHLH* gene family, we systematically investigated both tandem and segmental duplication events. Our analysis revealed 38 distinct tandem duplication clusters encompassing 91 genes (**Figure 5**). A representative example includes the five-gene cluster (*AsbHLH30-34*) localized on chromosome chr2A. Furthermore, we identified 152 segmental duplication pairs involving 160 *AsbHLH* members, revealing widespread genomic duplication contributions to family expansion. For instance, *AsbHLH001* and *AsbHLH011/014/077* were located on four different chromosomes: chr1A, chr1C, chr1D, and chr3A. These results indicate that duplication events are widespread among *AsbHLH* genes, that duplication events occur widely in *AsbHLH* genes, and that segmental duplications may be the driving force behind the evolution of the *AsbHLH* gene family.

**Figure 6:**
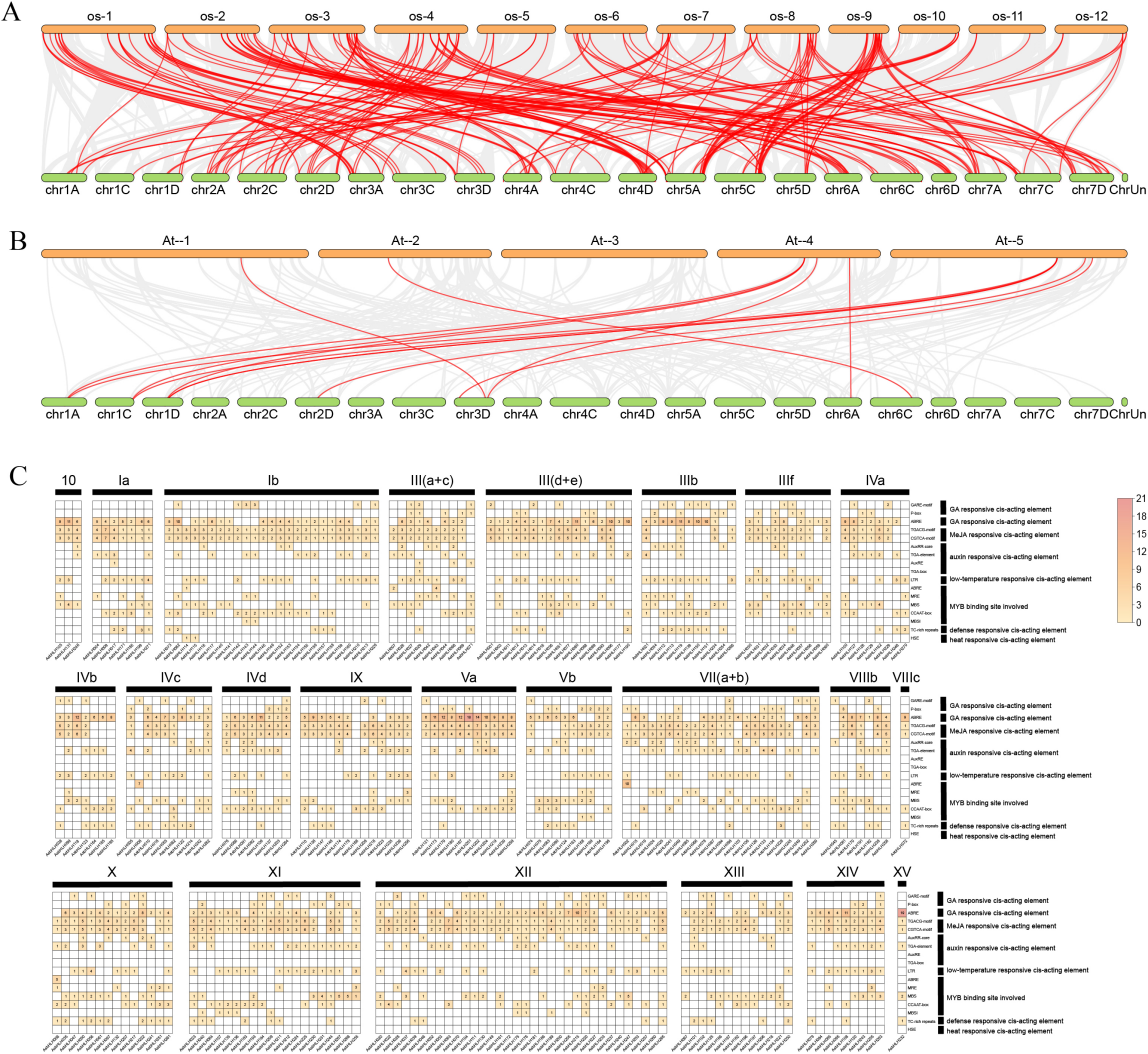
Collinearity analysis of *AsbHLH* genes with those from *A. thaliana* and *O. sativa*, and prediction of cis-acting elements in the promoters of *AsbHLH* genes. **(A)** Colinearity analysis of *bHLH* genes in rice and oat. Grey represents the homologous block in the genome, and red represents the duplication event of the fragment. **(B)** Colinearity analysis of *bHLH* genes in *Arabidopsis* and oat. Grey represents the homologous block in the genome, and red represents the duplication event of the fragment. **(C)** Prediction of cis-acting elements of oat *bHLH* gene promoter. The color depth indicates the number, and the number in the square also indicates the number.

To further explore the evolutionary relationships of the *AsbHLH* gene family and clarify potential evolutionary events of the *AsbHLH* gene family in various crops, covariance maps of oats with rice and *Arabidopsis* were constructed (**Figures 6A, B**). As shown, 124 *OsbHLHs* and eight *AtbHLHs* showed covariance with *AsbHLH* genes. Among these, *Arabidopsis* had 12 collinear gene pairs with oats, whereas oats and rice shared 193 gene pairs. The number of collinear genes between oats and rice was significantly higher than that between oats and *Arabidopsis*, revealing a closer evolutionary relationship between oats and rice in the *bHLH* gene family.

### 3.5 Analysis of cis-elements in the promoter regions of *AsbHLH* genes

To investigate the potential regulatory mechanisms of oat *AsbHLH* genes, we analyzed *cis*-acting elements within the 2-kb promoter regions upstream of their transcription start sites (**Figure 6C**). A total of 17 functionally distinct *cis*-elements were identified. The majority of *AsbHLH* promoters contained hormone-related motifs, including gibberellin-responsive elements (GARE-motif, P-box, and ABRE, which were present in 95.5% of members) and MeJA-responsive elements (TGACG/CGTCA, 85.9%). Auxin-responsive motifs (AuxRR-core, TGA-box, and TGA-element) were detected in 62.3% of promoters. This prevalence of hormone-related *cis*-elements indicates that *AsbHLH* genes likely play comprehensive roles in oat hormone signaling and stress adaptation. Furthermore, 83.7% of the members contained MYB-responsive elements (ABRE, MRE, MBS, CCAAT-box, and MBSI), 56.0% included low-temperature-responsive elements (LTR), 30.9% possessed defense-related cis-elements (TC-rich repeats), and 0.7% had heat shock-responsive elements (HSE). In addition, we observed that *AsbHLH* genes differ from other gene families in that no uniquely specific cis-acting elements were present in their promoter regions. These findings indicate that the expression of *AsbHLH* genes may be regulated by a combination of different hormonal and environmental stimuli.

### 3.6 Effects of different treatments on *AsbHLH* expression

**Figure 7:**
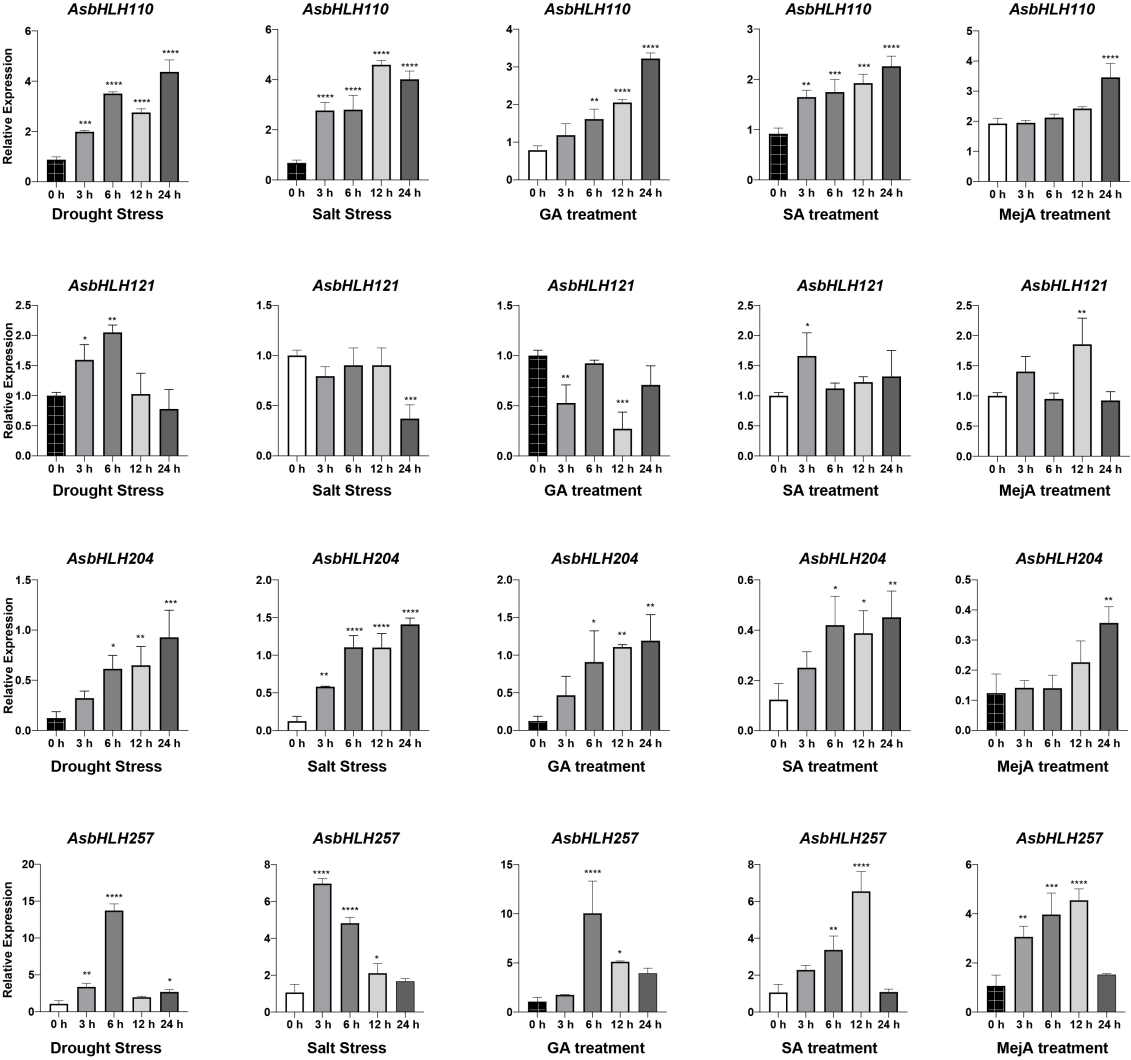
The expression of *AsbHLHs* in oat at different time under salt, drought, methyl jasmonate, salicylic acid and GA treatments. The error bar represents the standard error of the mean of three independent repetitions. One-way ANOVA revealed statistically significant differences. Significance levels: *P < 0.05; **P < 0.01, ***P < 0.001, ****P < 0.00001.

To investigate the role of *AsbHLH* genes during oat development, four representative genes, *AsbHLH110*, *AsbHLH121*, *AsbHLH204*, and *AsbHLH257*, were selected based on their homology to *AtbHLH* genes and the evolutionary relationship of the neighbor-joining tree. Their relative expression levels were assessed in oat leaves at various time points following different treatments using real-time quantitative reverse transcription-polymerase chain reaction (qRT-PCR) (**Figure 7**). As shown in **Figure 7**, *AsbHLH110*, *AsbHLH121*, *AsbHLH204*, and *AsbHLH257* were responsive to drought and salt stress. *AsbHLH110* and *AsbHLH257* expressions increased to varying degrees under MeJA, salicylic acid, and GA treatments. For *AsbHLH121*, expression peaked at 3 and 12 h following salicylic acid and MeJA treatment, respectively. *AsbHLH204* expression exhibited a continuous increase within 24 h in response to MeJA, salicylic acid, and GA. Overall, these expression patterns may reflect the distinct physiological pathways in which these genes participate.

### 3.7 The *bhlh257* mutant exhibits enhanced drought sensitivity and oxidative damage

**Figure 8:**
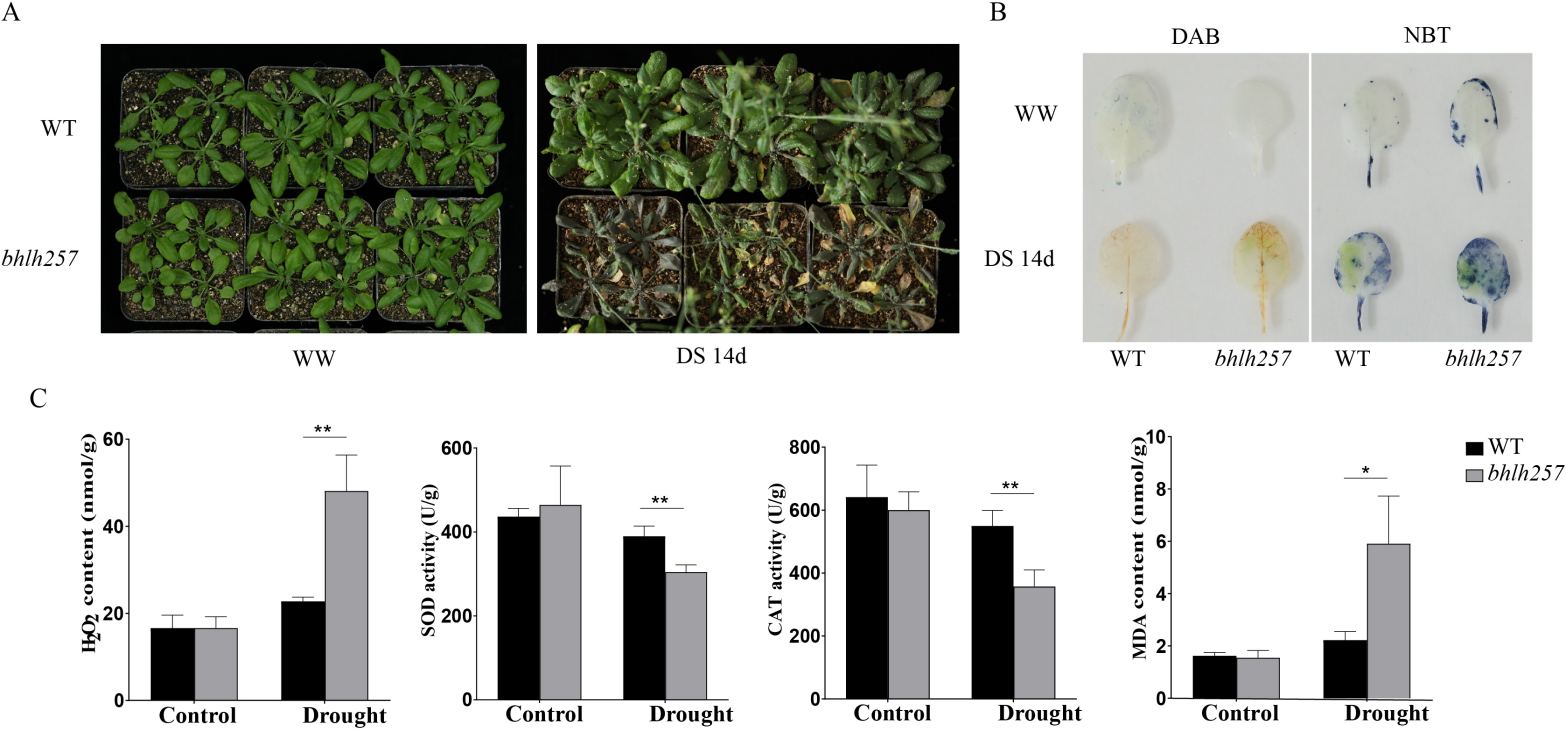
Physiological responses of *A. thaliana bhlh257* mutant to drought stress. **(A)** Phenotypes of wild-type (WT) and *bhlh257* mutant plants under well-watered conditions (WW) and drought stress treatment for 14 days (DS 14d). **(B)** Histochemical staining of leaves from control and drought-treated plants using DAB for *in situ* detection of H_2_O_2_ accumulation (brown precipitate) and NBT for detection of superoxide anion (O_2_^-^) accumulation (blue formazan precipitate). **(C)** Quantitative physiological indices of control and drought-treated plants. From left to right: H_2_O_2_ content (nmol/g), SOD activity (U/g), CAT activity (U/g), and MDA content (nmol/g). Asterisks indicate significant differences between wild-type and mutant under the same treatment (*P < 0.05, **P < 0.01; two-way ANOVA with Tukey’s *post-hoc* test).

To functionally validate a candidate gene from our prioritized list, we selected the Arabidopsis ortholog *bhlh257* mutant for further characterization. This gene was chosen because its expression was significantly induced under stress conditions in our preliminary screenings. To assess its function under physiologically relevant drought conditions, the mutant was analyzed during a 14-day soil drying cycle. Prior to stress treatment, both the mutant and WT plants were phenotypically indistinguishable. However, after exposure to drought conditions, while the WT plants remained green and turgid, the *bhlh257* mutants demonstrated severe wilting, chlorosis, and leaf purpling (**Figure 8A**). Histochemical staining revealed a higher degree of superoxide (NBT staining) and hydrogen peroxide (DAB staining) accumulation in drought-stressed mutant leaves as compared to that in the correspondingly treated WT leaves (**Figure 8B**). Quantitative assays confirmed a compromised oxidative stress response and elevated oxidative damage in the mutant, with the *bhlh257* plants accumulating significantly more H_2_O_2_ and malondialdehyde (MDA) than that in the WT plants under drought conditions (**Figure 8C**). Concurrently, the antioxidant enzymes superoxide dismutase (SOD) and catalase (CAT) were significantly less active in the stressed mutant as compared to those in the corresponding WT plants (**Figure 8C**). No genotypic differences were evident in well-watered plants. These data demonstrate that *bhlh257* is essential for drought tolerance, and its loss results in ROS overaccumulation, lipid peroxidation, and inadequate antioxidant defense.

## 4 Discussion

The bHLH transcription factor family represents one of the largest and most evolutionarily conserved gene families in eukaryotes, serving as key regulators in plant growth, development, and abiotic stress responses (Zuo et al., 2023). Although comprehensive genomic analyses of *bHLH* genes have been conducted in model plants (*Arabidopsis*) and major cereal crops (wheat, rice, and maize), the systematic characterization of this important transcription factor family remains unexplored in oats, an important global grain crop. Among the 271 AsbHLH proteins, DNA-binding activity was predicted based on the number of basic residues in the basic region of the *bHLH* domain (Toledo-Ortiz et al., 2003). Proteins containing at least five basic residues within this region were classified as DNA-binding bHLHs, whereas those lacking sufficient basic residues were considered non-binding HLHs (atypical bHLHs). According to this criterion, 57 (21.03%) *AsbHLH*s were predicted to possess DNA-binding activity, whereas 214 (78.97%) were classified as non-binding. Among the 26 *AsbHLH* subgroups, subgroup 8 was the most conserved, whereas subgroup 11 exhibited the greatest variability. This pattern is consistent with that observed in subgroups 8 and 11 of the *bHLH* families in the dicotyledonous plant *Arabidopsis* and monocotyledonous plant rice (Li et al., 2006). The greater variability in subgroup 11 indicates the absence of monocot-dicot specificity, suggesting that this subgroup in *AsbHLH* may have more divergent functional roles.

A phylogenetic tree constructed using the 271 AsbHLH proteins revealed that each AsbHLH subgroup contained at least one AtbHLH member, indicating that the bHLH family has diverged between arabidopsis and oats. Although the bHLH family size and subgroup organization show species-specific differences, these transcription factors consistently regulate core biological processes including development, stress responses, and evolutionary adaptation across plant lineages (Kang et al., 2016). For example, the significant expression of *bHLH* genes under abiotic stress has been observed in barley, thereby highlighting the importance of *bHLH* transcription factors in plant adaptation and development (Hernandez-Garcia and Finer, 2014; Ke et al., 2020). Moreover, the absence of significant clustering among *AsbHLH* genes suggests the possibility of a novel evolutionary trajectory in oats.

Gene structure analysis uncovered notable intron number variation within bHLH subgroups, with several members (such as *AsbHLH079*, *AsbHLH084*, and *AsbHLH095* in subgroup 14) exhibiting significantly reduced intron content compared to their subgroup counterparts. This structural simplification indicates potential evolutionary divergence in gene regulation mechanisms among paralogous genes. Notably, this intron reduction may have resulted from long-term evolutionary processes or environmental selection pressures (Yao et al., 2024). In plants, intron number is often correlated with gene expression levels, and a more compact gene structure may allow rapid transcriptional responses to environmental stimuli (Jeffares et al., 2008). Expansion of the *bHLH* gene family is primarily driven by gene duplication events (Carretero-Paulet et al., 2010). In oats, the *bHLH* family includes 12 tandemly duplicated clusters comprising 26 *AsbHLH* genes, as well as 95 segmentally duplicated gene pairs, revealing that segmental duplication has played a more prominent role in *bHLH* family expansion than tandem duplication.

The HLH gene family plays pivotal roles in regulating diverse physiological processes, including, plant growth and development, hormone signaling, and abiotic/biotic stress responses (Lei et al., 2024; Zeng et al., 2025). Promoter *cis*-element analysis of *AsbHLH* genes revealed an abundance of stress- and hormone-related regulatory motifs (e.g., ABRE, GARE-motif, and MeJA-responsive elements), thus further supporting their involvement in stress responses. The *Arabidopsis bhlh257* gene was characterized with the aim of functionally validating the role of *bHLH* genes in abiotic stress response, on the basis of its significantly differential expression under stress conditions in preliminary studies. The *bhlh257* mutant exhibited enhanced sensitivity to drought stress, including, severe wilting, chlorosis, and anthocyanin accumulation as compared to that in the wild-type plants. This phenotypic evidence directly demonstrates the essential function of *bHLH257* in drought tolerance.

At the mechanistic level, the hypersensitivity of *bhlh257* was associated with impaired oxidative stress management. The mutant accumulated excessive reactive oxygen species (ROS), as shown by intense NBT and DAB staining, along with elevated MDA content, indicating lipid peroxidation damage under drought conditions. Concurrently, the activities of key antioxidant enzymes, SOD and CAT, were significantly compromised in the mutant. These findings align with previous studies that demonstrate the regulation of abiotic stress responses, through the modulation of antioxidant defence systems by *bHLH* transcription factors (Dai et al., 2024).

Cross-species functional conservation supports the potentially critical role of *AsbHLH* genes in mediating stress adaptation in oats. The co-responsive expression pattern of selected oat *bHLH* genes (*AsbHLH110, AsbHLH121, AsbHLH204* and *AsbHLH257*) under multiple abiotic stresses and hormone treatments further suggests their involvement in integrated stress response networks. This is consistent with the established role of *bHLH* transcription factors as key regulators in plant stress signaling pathways (Kazan and Manners, 2013).

In oats, *bHLH* genes likely participate in ABA-mediated drought response pathways, similar to their orthologs in other plant species. For instance, while *AtbHLH122* enhances drought resistance in Arabidopsis by modulating ABA metabolism (W. Liu et al., 2014), *SbbHLH85* improves salt tolerance in sorghum by regulating root hair development (Song et al., 2022). These functional parallels across species strengthen the biological relevance of the oat *bHLH* candidates identified in this study.

Phytohormones are critical regulators of developmental pathways and stress-responsive signaling cascades in plants that facilitate adaptation to various biotic and abiotic stresses (Bari and Jones, 2009; Ding et al., 2025). The coordinated degradation of DELLA proteins and JASMONATE ZIM-domain (JAZ) repressors by gibberellins (GA) and jasmonates (JA) activates the WD-repeat/bHLH/MYB transcriptional complex, resulting in synergistic and interdependent trichome initiation that governs plant growth and development (Qi et al., 2014). *OsbHLH6* mediates disease resistance through nucleocytoplasmic shuttling mechanisms that modulate both salicylic acid (SA) and JA signaling pathways (Meng et al., 2020). In this study, cis-acting element analysis revealed that most of the identified elements were responsive to GA, MeJA, and MYB, and the expression of *AsbHLH110*, *AsbHLH204* and *AsbHLH257* was upregulated by varying degrees shortly post treatment with these three hormones.

This study provides the first comprehensive genome-wide analysis of the *bHLH* gene family in oat, thus establishing a valuable genetic resource for future research. The integration of phylogenetic analysis, gene duplication events, promoter cis-elements, and expression profiling has enabled the identification of promising candidate genes involved in abiotic stress responses. Further, the functional validation using the *Arabidopsis bhlh257* mutant further supports the biological significance of *bHLH* genes in drought tolerance. The present study is however limited by the fact that the molecular mechanisms underlying AsbHLH protein function, including, dimerization patterns and downstream target gene networks, remain unelucidated. Future studies that combine techniques such as yeast two-hybrid and ChIP-seq are necessary to address these questions.

## 5 Conclusions

This study presents the first genome-wide identification and characterization of the *bHLH* transcription factor family in hexaploid oat, thus establishing a key resource of the 271 *AsbHLH* genes that are unevenly distributed across all 21 chromosomes. Phylogenetic analysis revealed a high degree of identity with *Arabidopsis* and rice, alongside lineage-specific features, such as, the absence of the subgroup 20 orthologs. Gene family expansion was found to be primarily driven by segmental duplications. Expression profiling identified high-priority candidates, including *AsbHLH110*, *AsbHLH121*, *AsbHLH204*, and *AsbHLH257*, which were co-upregulated under salt and drought stresses, and modulated by multiple phytohormones (GA, MeJA, SA), thus indicating their potential roles as integrative hubs in stress-signaling networks. Functional validation using an *Arabidopsis bhlh257* mutant, an ortholog of the differentially expressed oat genes, confirmed the conserved role of *bHLH* transcription factors in drought tolerance. Further, the loss-of-function mutant exhibited enhanced drought sensitivity, coupled with excessive ROS accumulation and impaired antioxidant defense. In conclusion, this work establishes a prioritized candidate gene resource for the oat *bHLH* family, providing crucial evolutionary and expression-based evidence that narrow the targets for subsequent functional studies. It thus provides a valuable genetic resource with significant functional insights that will aid future molecular breeding efforts aimed at enhancing abiotic stress resilience in this economically crucial cereal.

## Data Availability

The original contributions presented in the study are included in the article/**Supplementary Material**. Further inquiries can be directed to the corresponding authors.
