# Supplementary figures and images for: Genome-wide identification and abiotic stress response analysis of the oat bHLH gene family
Source: Front Plant Sci. 2026 Apr 20;17:1832370. doi: 10.3389/fpls.2026.1832370 (PMC13137444; doi:10.3389/fpls.2026.1832370)

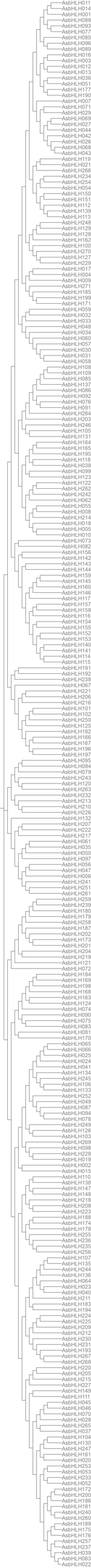

Supplement: Supplementary file 1 [file Image1.jpeg]
